# Attenuation of Dopaminergic Neurodegeneration in a *C. elegans* Parkinson’s Model through Regulation of Xanthine Dehydrogenase (XDH-1) Expression by the RNA Editase, ADR-2

**DOI:** 10.3390/jdb11020020

**Published:** 2023-05-22

**Authors:** Lindsey A. Starr, Luke E. McKay, Kylie N. Peter, Lena M. Seyfarth, Laura A. Berkowitz, Kim A. Caldwell, Guy A. Caldwell

**Affiliations:** 1 Department of Biological Sciences, Center for Convergent Biomedicine, Alabama Life Research Institute, The University of Alabama, Tuscaloosa, AL 35487, USA; 2 Department of Neurology, Center for Neurodegeneration and Experimental Therapeutics, Nathan Shock Center of Excellence for the Basic Biology of Aging, Heersink School of Medicine, University of Alabama at Birmingham, Birmingham, AL 35294, USA

**Keywords:** ABC transporter, α-synuclein, ADAR, *C. elegans*, dopamine, neurodegeneration, Parkinson’s disease, RNA editing, xanthine dehydrogenase

## Abstract

Differential RNA editing by adenosine deaminases that act on RNA (ADARs) has been implicated in several neurological disorders, including Parkinson’s disease (PD). Here, we report results of a RNAi screen of genes differentially regulated in *adr-2* mutants, normally encoding the only catalytically active ADAR in *Caenorhabditis elegans*, ADR-2. Subsequent analysis of candidate genes that alter the misfolding of human α-synuclein (α-syn) and dopaminergic neurodegeneration, two PD pathologies, reveal that reduced expression of *xdh-1*, the ortholog of human xanthine dehydrogenase (XDH), is protective against α-synuclein-induced dopaminergic neurodegeneration. Further, RNAi experiments show that WHT-2, the worm ortholog of the human ABCG2 transporter and a predicted interactor of XDH-1, is the rate-limiting factor in the ADR-2, XDH-1, WHT-2 system for dopaminergic neuroprotection. In silico structural modeling of WHT-2 indicates that the editing of one nucleotide in the *wht-2* mRNA leads to the substitution of threonine with alanine at residue 124 in the WHT-2 protein, changing hydrogen bonds in this region. Thus, we propose a model where *wht-2* is edited by ADR-2, which promotes optimal export of uric acid, a known substrate of WHT-2 and a product of XDH-1 activity. In the absence of editing, uric acid export is limited, provoking a reduction in *xdh-1* transcription to limit uric acid production and maintain cellular homeostasis. As a result, elevation of uric acid is protective against dopaminergic neuronal cell death. In turn, increased levels of uric acid are associated with a decrease in ROS production. Further, downregulation of *xdh-1* is protective against PD pathologies because decreased levels of XDH-1 correlate to a concomitant reduction in xanthine oxidase (XO), the form of the protein whose by-product is superoxide anion. These data indicate that modifying specific targets of RNA editing may represent a promising therapeutic strategy for PD.

## 1. Introduction

Parkinson’s disease (PD) is the second-most common neurodegenerative disorder, afflicting 1–2% of adults over age 65 worldwide [1]. PD is pathologically characterized by the progressive loss of dopaminergic neurons in the *substantia nigra pars compacta* (SNpc), accompanied by the misfolding of α-syn into beta sheets, forming Lewy bodies in the midbrain. Mitochondrial dysfunction is an important factor contributing to PD, occurring when there is an imbalance among reactive oxygen species (ROS) and antioxidants, resulting in cellular stress. Inhibition of complex I in the mitochondrial electron transport chain induces enhanced ROS generation [2], ultimately inhibiting activity of the proteasome, lysosomes, and mitochondria [3,4,5]. Further, enhanced cellular ROS promotes the misfolding of α-syn and perturbs protein clearance mechanisms, exacerbating disease [6]. Such pathologies have been found in the SNpc of PD patients [7,8].

Unfortunately, many hallmark symptoms of PD, such as bradykinesia, tremor, and abnormal gait, remain undetectable until a substantial amount of dopaminergic cell death has occurred [7]. Despite robust efforts to develop therapeutics for PD, treatment remains focused on the alleviation of symptoms, rather than ceasing disease progression. Moreover, the exact pathology underlying the foundation of this disease remains unclear. To date, twenty genes have been deemed causative of PD, while genome-wide association studies have identified 90 variants associated with PD risk [8]. However, the majority of PD cases are idiopathic, with disease spurring from interactions between genetic networks and environmental factors [9].

Adenosine to inosine (A-to-I) editing is the mechanism in which adenosine is converted to inosine in regions of double-stranded RNA; this base change is catalyzed by a family of enzymes known as adenosine deaminases that act on RNA (ADARs) [10]. The chemical properties of inosine are similar to those of guanosine, so it is treated as guanosine by translational machinery [11]. RNA editing by ADARs profoundly increases transcriptomic and proteomic diversity. ADARs are imperative for the healthy development and function of the nervous system [12], and ADAR protein dysfunction has been implicated in a variety of neurological diseases, including amyotrophic lateral sclerosis, epilepsy, developmental epileptic encephalopathy, and Aicardi-Goutières syndrome, among others [13]. Furthermore, knockouts of ADAR genes in model organisms have exhibited profound neurological dysfunction. For example, ADAR2 knockout mice are prone to seizures and have a shortened lifespan [14], and *Adar* mutant *Drosophila* display synaptic and neurotransmission defects [15]. Notably, a recent study in post mortem brain tissue revealed differential editing in mRNA of PD patients that succumbed to disease [16]. Thus, it is plausible that ADARs have a salient role in PD.

The nematode roundworm, *C. elegans*, is an exceptional model organism to study progressive neurodegenerative diseases, such as PD, due to its hermaphroditic reproduction, large brood size, short generation time (~three days), and ease of maintenance [17,18]. Additionally, the entire connectome of *C. elegans* has been delineated [19]. Thus, *C. elegans* provides researchers with the ability to rapidly analyze isogenic populations and precisely quantify neurodegeneration in individuals disadvantaged by α-syn, the central protein associated with dopaminergic neurodegeneration in PD. In *C. elegans*, two ADAR genes with human orthologs have been identified, *adr-1* and *adr-2*. Analyses of these genes indicated that both have distinct roles in RNA editing and are essential for normal nervous system function in *C. elegans* [20]. However, ADR-2 is the only active adenosine deaminase in *C. elegans*, as knockout of *adr-2* abolishes all A-to-I RNA editing [21]. In contrast, ADR-1 acts as a regulator of ADR-2 by facilitating the binding of ADR-2 to its targets [21,22].Comparative high-throughput sequencing of the transcriptomes of wildtype vs. *adr-2* mutants has resulted in the identification of thousands of differentially edited sites in *C. elegans* RNAs that represent putative changes impacting the expression, stability, or function of specific gene products [23]. This research focuses on two evolutionarily conserved genes, *xdh-1* and *wht-2*, that are regulated by *adr-2*-dependent expression and editing, respectively, in *C. elegans*.

Xanthine dehydrogenase (XDH) is an enzyme that is responsible for the catabolism of hypoxanthine to xanthine and xanthine to uric acid, the final two steps in the purine catabolic pathway [24,25]. Located intracellularly in peroxisomes and the cytosol, XDH has two subunits, each consisting of four redox center domains: a molybdopterin center, two iron sulfur centers, a flavin adenine dinucleotide (FAD) center, and two iron–sulfur subunits [26]. NADH and uric acid are produced as byproducts of XDH activity. Additionally, XDH can be converted to xanthine oxidase (XO), both irreversibly and reversibly, via proteolysis or the oxidation of two cysteine residues, respectively. This interconversion occurs in the presence of urea or when concentrations of guanidine hydrochloride are low. Oxygen is required for XO activity in lieu of NAD, yielding superoxide anion, hydrogen peroxide, and uric acid [26]. This difference in cofactors is attributed to the capacity for XDH to rapidly react with NAD and to react slowly with oxygen, while XO does the opposite [27]. This stems from the conformational change that results upon XDH to XO conversion—in the simplest terms, the XDH has a higher binding affinity than XO [26]. As a result, XO is a major generator of ROS (superoxide anion and hydrogen peroxide), which significantly contribute to oxidative stress.

ATP binding cassette subfamily G member 2 (ABCG2), the human ortholog of worm WHT-2, is an efflux transporter containing a hydrophobic transmembrane domain and a nucleotide binding domain [28]. It can be found in various tissues, such as the intestine, kidney, and brain. Specifically, it is expressed on the apical side of endothelial cells in the blood–brain barrier (BBB). It has also been shown to be located in mitochondrial cristae [29]. ABCG2 is a transporter of many endogenous substrates, such as urate, steroids, and heme, as well as xenobiotic compounds. Importantly, it has been shown to be upregulated in response to oxidative stress, as it is able to efflux substances that generate ROS [30].

The spatial and temporal landscape of ADAR-dependent regulation is vast, and largely uncharted with respect to its effects on normal biological processes and in disease. The dynamic nature of RNA editase activity is intriguing and represents an untapped area of potential therapeutic targeting for PD. Here, we provide experimental evidence that show a reduction in functional XDH in dopaminergic neurons is protective against neurodegeneration, attributed to oxidative stress induced by α-syn. Further, we propose a network in which ADR-2 edits WHT-2/ABCG2, altering its structure and its ability to export uric acid from the cell. In turn, XDH is downregulated to maintain homeostasis and prevent potential deleterious effects of excess uric acid. As a result, downregulation of *xdh-1* promotes the folding of α-syn into a less cytotoxic conformation and enhances dopaminergic neuroprotection, since reduced levels of XDH simultaneously represent a decrease in XO, leading to a decrease in ROS production.

## 2. Materials and Methods

### 2.1. C. elegans Strains

All strains were maintained according to standard procedures [31]. For RNAi experiments, UA44 (P*_dat-1_*::α-syn, P*_dat-1_*::GFP) and UA49 [P*_unc-54_*::α-syn::GFP, *rol-6* (*baIn2*)] were used to knockdown genes in all tissues, excluding neurons, which are inherently resistant to RNAi due to low expression levels of the *sid-1* gene, encoding the SID-1 dsRNA transporter needed to confer systemic RNAi sensitivity. UA196 {*sid-1(pk3321*);P*_dat-1_*::*sid-1*, P*_myo-2_::*mCherry; P*_dat-1_*::GFP, P*_dat-1_*::α-syn} is a specialized RNAi-sensitive strain, previously generated in our lab to facilitate the knockdown of genes in dopaminergic neurons only by the selective dopaminergic expression of *sid-1* in mutant animals that are otherwise depleted of *sid-1* {*sid-1(pk3321*)} [32,33].

Three stable lines of worms, overexpressing *xdh-1*, driven by the dopamine transporter (*dat-1*) promoter, were generated by injecting both the P*_dat-1_*::*xdh-1* construct (kindly gifted by Atsushi Kuhara) and *rol-6* co-injection marker at a concentration of 50 ng/μL each (100 ng/μL total) into UA44 worms. The resulting genotype of each of the three stable lines was P*_dat-1_*::*xdh-1; vtIs7*[P*_dat-1_*::α-syn, P*_dat-1_*::GFP].

Other strains used in this study include RB2379 *(xdh-1(ok3134))* and RB886 *(adr-2(ok735))*. BY250 (*vtIs7*[P*_dat-1_::*GFP]), kindly provided by Randy Blakely, as well as UA457 (*adr-2(ok735)*; [P*_dat-1_*::α-syn, P*_dat-1_*::GFP]). Animals with the *adr-2(ok735)* mutant allele were a gift from Heather Hundley. Genetic crosses to generate new worm strains used in this study are summarized in Table 1.

### 2.2. RNAi and Analysis of Protein Misfolding

RNAi by feeding was performed, as described [34]. Briefly, bacteria containing a plasmid dsRNA of the gene of interest, generated using the L4440 plasmid vector, were fed to target worms. A control group was fed bacteria with just the plasmid L4440, known as an empty vector (EV). UA49 worms were fed RNAi bacteria, and individual animals were scored semi-quantitatively for aggregate size and number two days post hatch. For our purposes, aggregates were defined as visible clumps of misfolded α-syn. Two-day old worms were analyzed because, at this age, *C. elegans* do not yet produce ovum; once ova are produced, the body wall muscles are thin and are not suitable for scoring. For each gene, two replicates of forty worms each were immobilized with 2 mM levamisole, transferred onto an agarose pad, and they were analyzed via fluorescent microscopy. The body wall muscles in the center of the worm were analyzed in all animals. The sizes of misfolded proteins were categorized as small, medium, or large, and the number of aggregates were denoted as few, multiple, or many. These categories were then quantified by multiplying by 1 for small or few, 2 for medium or multiple, and 3 for large or many to obtain a scaled score for size and number. These scores were added together to obtain an overall aggregate score, and the percent difference of each experiment compared to the empty vector (EV) control was determined.

### 2.3. GO Term Analysis of Candidate Genes

Gene enrichment analysis was performed to identify tissues, phenotypes, and gene ontology (GO) terms associated with the annotations of candidate genes. All analyses were performed using the WormBase enrichment suite [34].

### 2.4. Analysis of Neurodegeneration

RNAi by feeding was performed [35] in UA44 and UA196 worms, and animals were scored for neurodegeneration on day 7, post hatch. Knockdown in UA44 leads to reduced gene expression in all tissue types, excluding neurons, while RNAi in UA196 results in knockdown in dopaminergic neurons only. Worms were transferred to fresh plates on days 4, 5, and 6 to ensure that only target worms were analyzed and not confused with progeny. Three replicates of 30 worms each were immobilized with 2 mM levamisole, transferred onto an agarose pad, and analyzed via fluorescent microscopy. The four CEP and two ADE neurons of each worm were analyzed for the presence of a cell body and intact axon process. Worms with all six neurons intact were considered wildtype, and those with one or more degenerated neurons were noted as degenerative. Worms were also grouped by the number of degenerated neurons [36]. The same experimental paradigm was used to analyze dopaminergic neuronal health in UA44 and UA455 ((P*_dat-1_*::α-syn and P*_dat-1_*::α-syn + *xdh-1(ok3134*)). However, for non-RNAi experiments, worms were grown on OP50-1 bacteria.

Each *of* the three stable lines of worms overexpressing *xdh-1* under control of the P*_dat-1_* promoter in a UA44 background (P*_dat-1_*:: *xdh-1*; *vtIs7*[P*_dat-1_*::α-syn, P*_dat-1_*:: GFP]) were analyzed. Specifically, three replicates, consisting of 30 worms of each of three stable lines, were analyzed, as described above; however, worms were scored 4 days post hatch. This day was chosen because our neuronal PD worm models exhibit time-dependent neurodegeneration, and we have observed that enhanced neurodegeneration is best observed in younger adult worms. Data from analysis of three replicates from each of the three stable lines (Figure S3) were averaged and considered as one biological replicate for statistical purposes.

### 2.5. Body Wall Muscle and Dopaminergic Neuron Image Acquisition

Images of worm body wall muscles and dopaminergic neurons were obtained by placing worms in a 7 μL drop of 10 mM levamisole (dissolved in 0.5× S basal buffer) on a glass coverslip. The coverslip with the worms in levamisole was inverted and placed on a 2% agarose pad attached to a microscope slide, paralyzing worms to allow for visualization. Fluorescence microscopy was performed with a Nikon Eclipse E800 epifluorescence microscope (Nikon Instruments Inc., Melville, NY, USA) equipped with an Endow GFP HYQ filter cube. Images were captured using a Cool Snap CCD camera (Teledyne Photometrics, Tuscon, AZ, USA) with Metamorph software (Molecular Devices LLC, San Jose, CA, USA).

### 2.6. RT-qPCR of α-syn Expression

Total RNA from UA44 and UA455 worms was isolated seven days post hatching. RNA from 100 worms from three replicates of each group was isolated using TRI reagent (Molecular Research Center Inc., Cincinatti, OH, USA). Samples were rid of genomic DNA contamination with 1 μL of DNaseI (Promega Corp, Madison, WI, USA) treatment for 60 min at 37 °C, then with DNase Stop solution for 10 min at 65 °C. An amount of 1 μg of RNA was used for cDNA synthesis using the iScript Reverse Transcription Supermix for RT-qPCR (Bio-Rad Laboratories Inc., Hercules, CA, USA), following the manufacturer’s protocol. RT-qPCR was performed using IQ-SYBR Green Supermix (Bio-Rad) with the Bio-Rad CFX96 Real-Time System. Reactions consisted of 7.5 μL of IQ SYBR Green Supermix, 200 nM of forward and reverse primers, and 5 ng of cDNA, to a final volume of 15 μL. Following thermocycling, a melting curve analysis was performed using the default setting of the CFX96 Real-Time System. Single melt peaks were observed for each targeted gene. The PCR efficiency for each primer pair was calculated from standard curves generated using serial dilutions: Eα-syn = 99.0%, E*snb-1* = 99.8%, E*tba-1* = 99.5%, and E*ama-1* = 103.1%. The expression levels of α-syn were normalized to three reference genes: *snb-1*, *tba-1*, and *ama-1*. No template control (NTC) and no reverse transcriptase control (NRT) exhibited amplification. All reference genes used were analyzed by geNorm (www.genorm.cmgg.be, accessed on 8 December 2022) and passed for target stability. Three independent biological replicates, with three technical replicates each, were tested for both worm strains. Data analysis was executed using the “do my qPCR calculation” web tool [37]. The following primers were used:

α-syn Forward: 5′-ATGTAGGCTCCAAAACCAAGG-3′
α-syn Reverse: 5′-ACTGCTCCTCCAACATTTGTC-3′
*snb-1* Forward: 5′-CCGGATAAGACCATCTTGACG-3′
*snb-1* Reverse: 5′-GACGACTTCATCAACCTGAGC-3′
*tba-1* Forward: 5′-ATCTCTGCTGACAAGGCTTAC-3′
*tba-1* Reverse: 5′-GTACAAGAGGCAAACAGCCAT-3′
*ama-1* Forward: 5′-TCCTACGATGTATCGAGGCAA-3′
*ama-1* Reverse: 5′-CTCCCTCCGGTGTAATAATGA-3′

### 2.7. Quantification of ROS

A DCF-DA assay was completed with N2 (wildtype), *xdh-1(ok313*4), UA44 [P*_dat-1_*::α-syn], and UA455 [P*_dat-1_*::α-syn + *xdh-1(ok3134*)] animals seven days post hatch. An acetylated form of fluorescein that is able to diffuse into the cell was used; once in the cell, the acetate groups of this fluorescein are removed by cellular ROS, producing a fluorescent end-product [38]. Worms were washed three times prior to the start of the experiment. The assay was completed using a microplate reader, shaking at 37 °C. An amount of 50 μL of M9 and 50 μL of 100 μM DCF-DA (ThermoFisher Scientific, Waltham, MA, USA) were added to each sample for a final concentration of 50 μM DCF-DA. Plate spectroscopy readouts were recorded at an excitation of 485 nm and an emission of 525 nm every 15 min for a total of 2.5 h. Normalization of DCF-DA signal-to-cell population was conducted prior to the start of the assay. Three biological replicates and two technical replicates were completed for each worm strain.

### 2.8. In Silico Stuctural Modeling of the WHT-2 Protein as Predicted for Unedited vs. Edited wht-2

Previously, two editing events in the *wht-2* gene (IV: 11470944 and IV: 11472326), corresponding to nucleotides that are edited by ADR-2, were identified in mRNA from *C. elegans* neural cells [39]. To determine the impact of editing at these sites, adenosines were changed to guanosines, since translational machinery treats inosines as guanosines. Using EMBOSS [40], the modified RNA sequence that reflects the impact of editing was translated in silico, and the edited and unedited amino acid sequences were aligned to determined differences between the two sequences. *C. elegans wht-2* and human ABCG2 were also aligned using EMBOSS to determine whether the identified editing site is conserved in humans. AlphaFold [41] and ChimeraX [42] were used to visualize the impact of this amino acid change on the WHT-2 protein.

### 2.9. Xanthine Oxidase Activity and Uric Acid Assays

For both assays, RNAi by feeding of EV, *adr-2*, *xdh-1*, *wht-2*, and *xdh-1 + wht-2* was performed in UA49 [P_unc-54_::α-syn::GFP, *rol-6 (baIn2)*] worms. An amount of ~200*–*250 two-day old worms were washed 3 times with 0.5*×* M9. Worms were frozen and thawed three times, and, subsequently, they were sonicated using an Ultrasonic Processor GEX 130PB (Daigger Scientific Inc., Hamilton, NJ, USA), for three cycles of 10 s on and 30 s off at a 40% amplitude. Samples were spun down at 4 °C and 15,000× *g* for 10 min. An amount of 50 µL of sample was loaded into a 96-well plate. Reagents for each assay were prepared according to their respective kit [(Xanthine Oxidase Activity Kit Calorimetric Analysis, Sigma-Aldrich Inc., St. Louis, MI, USA) (Uric Acid Assay Kit Fluoremetric Analysis, Cell BioLabs Inc., San Diego, CA, USA)]. Experiments were performed according to kit instructions, and reads were taken using a Spectraax M2e Microplate Reader every 5 min for 1 h. For the XO assay, measurements were taken at 570 nm. For the uric acid assay, measurements were taken at an excitation of 550 nm and an emission of 585 nm.

### 2.10. Statistical Analysis

For all data analyses, the mean and standard error of the mean (SEM) were determined, and the data were analyzed via the chi-square test, unpaired T test, one-way ANOVA with Tukey’s post hoc test, or two-way ANOVA with Sidak’s post hoc test (GraphPad Prism, version 8.0.1, accessed on 28 February 2023). Values below 0.05 were considered significant.

## 3. Results

### 3.1. Genes Regulated by ADR-2 Alter α-syn Misfolding

This investigation began with the hypothesis that RNA editing by ADARs has a role in PD. In this study, we used two published gene sets: (1) sites edited in *C. elegans* neural cells in worms and (2) RNAseq of neural cells in wildtype vs. *adr-2* mutant worms [43]. The investigation began by knocking down genes differentially expressed in neural cells of *adr-2* mutants because it allowed us to experimentally analyze the functional impact of editing directly by ADR-2. The editing of genes does not typically lead to a change in gene expression of the edited gene in the *C. elegans* neural editome; in fact, in the two previously published datasets, only four genes are edited by ADR-2 in worm neural cells, and they are also differentially regulated in neural cells of *adr-2* mutants [43]. This is likely a result of most editing sites occurring in intronic regions or 3′ UTRs. We reasoned that screening ADR-2 targets via RNAi would not reflect ADR-2 activity because editing typically results in the change in bases in pre-mRNA regions that are more complex to analyze. Thus, we discerned that we were more likely to identify a gene by RNAi whose expression is regulated by ADR-2 that impacts PD pathologies; we could more thoroughly investigate such a modifier and then connect it to a target that is edited by ADR-2.

**Figure 1:**
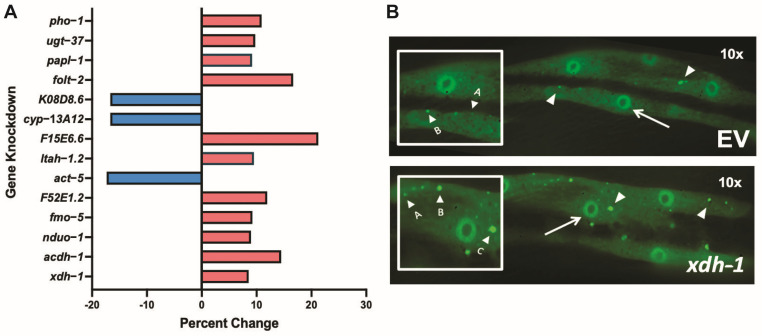
RNAi of genes differentially regulated in *adr*-*2* mutants alter protein misfolding. (**A**) Isogenic worm strain expressing α-syn::GFP in the body wall muscle cells of *C. elegans* were fed *E. coli* expressing candidate gene RNAi constructs and scored for aggregate size and number, which were combined to generate an overall aggregate score. Synchronized worms were analyzed two days post hatch, and a percent change for each gene was calculated in comparison to empty vector control. The top 14 genes that resulted in the greatest cumulative percent change in aggregation are represented. (**B**) RNAi of *xdh*-*1* (**bottom**) in worms expressing α-syn::GFP in the body wall muscles under control of the P*_unc-54_* promoter showed increased aggregate size and number in comparison to empty vector control (**top**). Images were taken at 10× and modified to best display the entire body wall muscle cell and their features. Examples of scoring criteria: A = small aggregate, B = medium aggregate, C = large aggregate, arrow = nuclei of body wall muscle cell, arrowhead = representative aggregates.

An amount of 169 genes were previously reported to be up- or down-regulated in neural cells of *C. elegans* mutant for *adr-2*; thus, expression of these genes is regulated by ADR-2 [25]. To identify presumptive effectors of the accumulation and misfolding of α-syn, genes regulated by ADR-2 with human orthologs were analyzed in an isogenic *C. elegans* strain, in which α-syn and GFP are fused and co-expressed in the body wall muscle, the largest cell type in this species [44]. In such worms, misfolded α-syn is evident in the body wall and increases or decreases in aggregated α-syn can be easily evaluated. All genes with human orthologs were screened via RNAi in two-day old worms. Forty worms were subjected to RNAi in duplicate for each evaluated gene, and the body walls of individual worms were analyzed for aggregate size and number (Figure 1 and Table 2). The fourteen genes that resulted in the greatest change in aggregation were considered strong candidates for further examination. Among these genes, only *F52E1.2* and *fmo-5* are upregulated in the *adr-2* mutant—all other genes are downregulated [43].

### 3.2. Modifiers of Protein Misfolding Are Associated with FAD and Iron Binding

**Figure 2:**
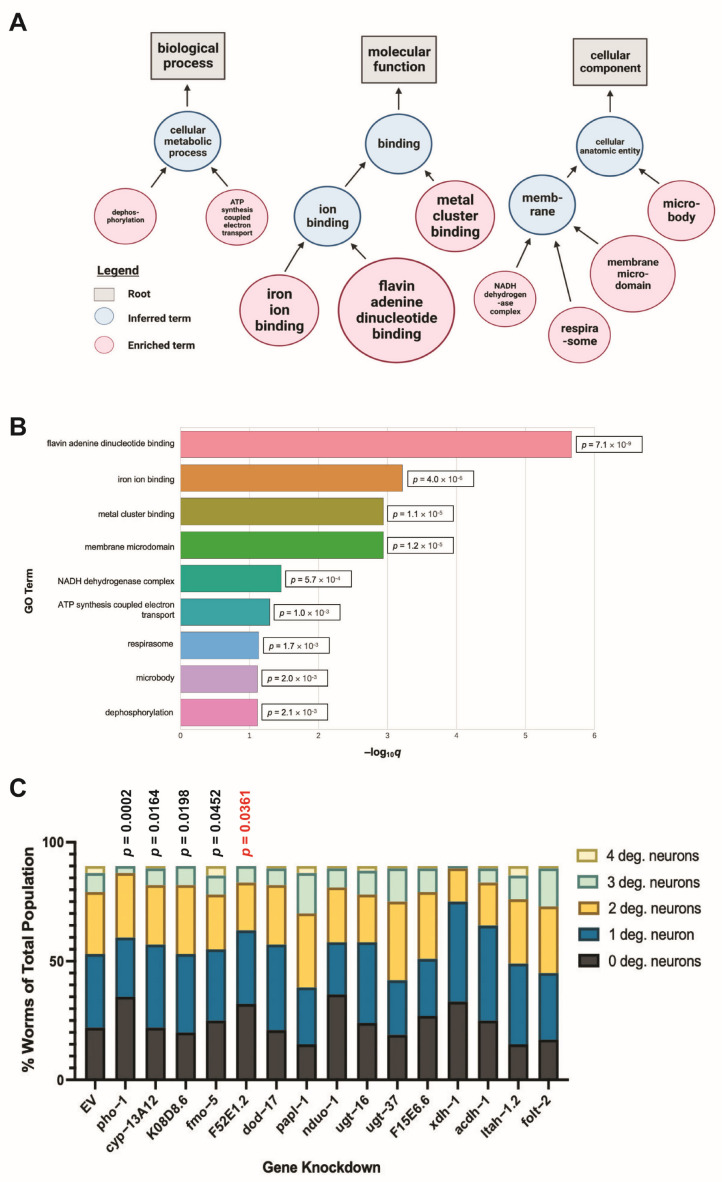
ADR-2-regulated candidate modifiers of α-syn misfolding are most highly associated with FAD and iron ion binding and impact dopamine neuronal health. (**A**) Graphical representation of biological processes (www.wormbase.org, accessed 25 July 2022), molecular functions, and cellular components associated with genes differentially regulated in *adr-2* mutants and which alter α-syn misfolding. Grey boxes indicate root term. Red circles represent terms overrepresented in the gene set, while blue circles designate inferred terms to connect the enriched terms. (**B**) GO terms enriched in candidate genes. (**C**) Candidate genes were knocked down in dopaminergic neurons of C. elegans expressing α-syn and GFP under control of the P_dat_*_-_*_1_ promoter. Subsequent scoring of dopaminergic neuronal health and worm population analysis indicate that RNAi of *xdh*-*1*, *acdh*-*1*, *pho*-*1*, and *F52E1.2* were protective against α-syn-induced dopaminergic neurodegeneration, while *papl*-*1* knockdown enhanced dopaminergic neuronal death. EV indicates empty vector control. Worms were analyzed as seven-day old adults. Chi-square analysis was performed, where each candidate gene knockdown value was compared to the empty vector (EV), and each gene is represented by exact *p* values (three independent experiments, *n* = 30 per independent groups; *N* = 3; *n* = 30).

To functionally analyze the fourteen identified candidates, a gene enrichment analysis was performed to identify highly associated biological pathways, molecular mechanisms, and phenotypes (Figure 2A). Of these, the two most significant terms were flavin adenine dinucleotide (FAD) binding and iron ion binding (Figure 2B)—four genes were affiliated with FAD binding, and three were connected to iron binding.

### 3.3. Select ADR-2-Regulated Modifiers of Protein Misfolding Impact Neurodegeneration When Knocked Down in Dopamine Neurons

Other significant processes connected to these candidates include function of the NADH dehydrogenase complex, ATP synthesis-coupled electron transport, and respirasome activity—all affiliated with the electron transport chain (Figure 2A). Since these genes are so strongly correlated with PD pathologies and altered protein-misfolding of α-syn, it was hypothesized that they likely represent functional effectors of dopaminergic neurodegeneration. To determine the impact of the candidate genes on dopaminergic neuronal death, RNAi of each candidate gene was performed in a RNAi-sensitive worm strain in which knockdown is delimited to only the dopamine neurons—these worms also overexpress WT human α-syn and GFP, independently, both under the control of the dopamine transporter (P*_dat-1_*) promoter and which allow for age- and dose-dependent analysis of neurodegeneration [34]. Following treatment, the four cephalic (CEP) and two anterior deirid (ADE) dopaminergic neurons of all worms in each population were analyzed via fluorescent microscopy, and individual worms were subsequently categorized as having zero degenerated neurons, one degenerated neuron, two degenerated neurons, three degenerated neurons, or four to six degenerated neurons. This experimental paradigm facilitated the examination of the neuronal health of the entire population of worms in each group (Figure 2C). Of the candidates, one gene, *papl-1*, enhanced neurodegeneration, while four, *xdh-1*, *acdh-1*, *pho-1*, and *F52E1.2*, were protective when knocked down (Table 3). RNAi targeting of *xdh-1*, *pho-1*, or *F52E1.2* resulted in an increase in animals with six wildtype anterior dopamine neurons, suggesting that reduced expression of these genes supports dopaminergic neuronal health. RNAi of *acdh-1* did not lead to more animals having zero degenerated neurons (wild-type); however, more individuals in this group displayed one degenerated neuron in comparison to the EV control. Because RNAi only leads to reduced gene expression, these data indicate that complete knockout of *acdh-1* may be neuroprotective against α-syn-induced neurodegeneration.

Of the four protective genes, *xdh-1*, the ortholog of human XDH, was the most significant. Furthermore, all the genes in which a decrease in expression led to a neuronal phenotype function in enzymatic activity. Of the candidates which were neuroprotective on knockdown, *xdh-1* had the strongest protective effect. Additionally, RNAi of candidate genes was performed in worms expressing α-syn and GFP, separately, in dopaminergic neurons (UA44), where knockdown occurs in all tissue types, excluding neurons (Figure S1). *xdh-1* was not protective in this strain, indicating that the protective phenotype is tissue-specific, and a decrease in gene expression must occur in dopamine neurons for protection to result.

### 3.4. Loss of XDH Leads to Neuroprotection through the Reduction in ROS

**Figure 3:**
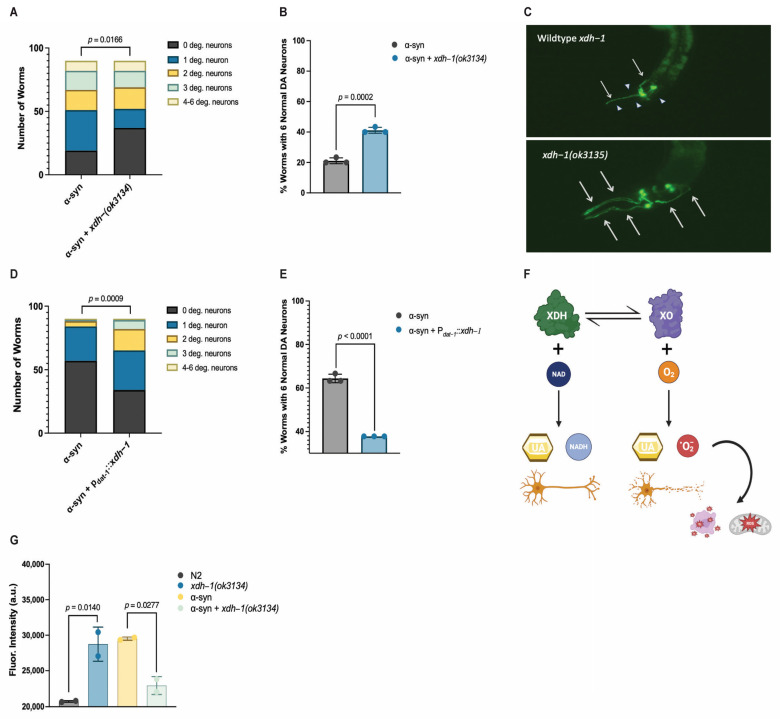
XDH-1 modulates neuroprotection by impacting ROS production. (**A**,**B**) Animals with wildtype or mutant *xdh-*1 were crossed to isogenic worms expressing α-syn + GFP in dopamine neurons. The four CEP and two ADE dopaminergic neurons were analyzed for the presence or absence of intact cell bodies and axonal processes in synchronized worms seven days post hatch (three independent experiments, *n* = 30 per independent groups). Worms with the *xdh-1(ok3134)* mutation exhibit neuroprotection against α-syn. (**A**) The number of degenerated neurons in each individual worm of the total population. Chi-square analysis was performed where α-syn + *xdh*-*1(ok3134)* was compared to α-syn alone and values are represented by exact *p* values (three independent experiments, *n* = 30 per independent groups; *N* = 3; *n* = 30). (**B**) The percentage of worms with six normal dopaminergic neurons. Student’s *t*-test, where α-syn + *xdh*-*1(ok3134)* was compared to α-syn alone, and values are represented by exact *p* values (three independent experiments, *n* = 30 per independent groups; *N* = 3; *n* = 30). (**C**) *C. elegans* expressing α-syn + GFP in dopaminergic neurons, wildtype, and mutant for *xdh-1*. Arrows represent wildtype dopamine neurons with a cell body and intact axonal process, and arrowheads represent degenerated dopamine neurons. The *xdh-1* mutation confers protection against α-syn induced neurodegeneration. (**D**,**E**) Worms overexpressing *xdh-1* under control of the P*_dat_-_1_* promoter were crossed with worms expressing α-syn + GFP in dopaminergic neurons. Subsequently, animals were synchronized and scored for neurodegeneration four days post hatch. This time point was chosen because worms expressing α-syn + GFP in dopaminergic neurons exhibit time-dependent neurodegeneration, and we have observed that enhanced neurodegeneration is more readily detected in younger adult worms. Worms with increased *xdh-1* expression in dopaminergic neurons showed decreased neuroprotection in four-day old worms. (**D**) The number of degenerated neurons in each individual worm of the total population. Chi square analysis was performed, where α-syn + P*_dat-1_*::*xdh-1* was compared to α-syn alone, and values are represented by exact *p* values (three independent experiments, *n* = 30 per independent groups; *N* = 3; *n* = 30). (**E**) The percentage of worms with six normal dopaminergic neurons. Student’s *t*-test where α-syn + P*_dat-1_*::*xdh-1* was compared to α-syn alone and values are represented by exact *p* values (three independent experiments, *n* = 30 per independent groups; *N* = 3; *n* = 30). (**F**) In the purine catabolic pathway, xanthine dehydrogenase (XDH) and xanthine oxidase (XO) catalyze hypoxanthine to xanthine and xanthine to uric acid. XDH and XO are interconvertible and are encoded by a single gene. For XDH activity, NAD is required as a cofactor, and NADH and uric acid are produced. In contrast, molecular oxygen is needed for XO to function, and uric acid and superoxide anions result. Reactive oxygen species (ROS) contribute to mitochondrial dysfunction, oxidative stress, and ultimately cell death. Created with Biorender.com. (**G**) Wildtype N2 worms, *xdh*-*1(ok3134)* mutant animals, and transgenic worms expressing α-syn in dopaminergic neurons with and without the *xdh*-*1(ok3134)* mutant, were treated with a specialized fluorescein to quantify ROS in the sample. Three biological replicates and two technical replicates were completed for all worm strains. After 2.5 h, worms expressing α-syn and GFP in dopaminergic neurons that are mutant for *xdh-1* produce lower ROS than worms without the mutation. Significance was obtained using a One-way ANOVA with a Tukey’s post hoc test and are represented by exact *p* values (*N* = 3; *n* = 30).

To further explore the relationship between *xdh-1* and neuroprotection, worms overexpressing GFP and α-syn under the control of the DA neuron-specific *dat-1* promoter were crossed to *xdh-1* mutants. *xdh-1* mutants displayed substantial protection against α-syn-induced neurodegeneration seven days post hatch (Figure 3A–C). In fact, the number of *xdh-*1 mutants with six wildtype dopamine neurons was nearly double that of worms lacking the mutation. qPCR of α-syn was performed to ensure that the observed neuroprotection was not due to silencing of the α-syn transgene (Figure S2). Additionally, *xdh-1* mutants were crossed to worms expressing GFP alone under control of the *dat-1* promoter; these worms did not display enhanced dopamine neurodegeneration (Figure S3). Thus, knockout of *xdh-1* is robustly neuroprotective against α-syn toxicity, but it is not detrimental to dopamine neuronal health in the absence of α-syn. To determine the impact of increased *xdh-1* expression on dopaminergic neuronal health, a transgenic worm strain in which dopaminergic overexpression of α-syn, GFP, and *xdh-1* was generated and subsequently analyzed for neurodegeneration four days post-hatch. This time point was chosen for analysis because worms that express α-syn and GFP in dopaminergic neurons exhibit time-dependent neurodegeneration, and we have observed that enhanced neurodegeneration is best shown in younger adult worms. These worms exhibited significantly more neurodegeneration than worms only expressing α-syn and GFP in dopaminergic neurons four days post-hatch (Figure 3D,E).

Although *xdh-1* mRNA is translated to form XDH, it regularly interconverts post-translationally to XO. This conversion to an alternate enzymatic product may occur reversibly through the oxidation of cysteine to form disulfide bridges, or irreversibly via proteolysis [26,48]. While the final product of purine catabolism by both XDH and XO is uric acid, XDH activity also generates NADH, whereas XO function yields ROS (Figure 3F). Thus, it is plausible that worm mutants for *xdh-1* exhibit neuroprotective phenotypes due to a reduction in XO-associated ROS production. To confirm this possibility, an assay to measure ROS was used in wildtype and mutant *xdh-1* worms either expressing or not expressing α-syn in the *C. elegans* dopamine neurons. Indeed, worms harboring a deletion knockout in the *xdh-1* locus exhibited a substantial decrease in ROS, conceivably leading to neuroprotection (Figure 3G). The opposite was found in animals not expressing α-syn, in that *xdh-1(ok3134)* mutants displayed enhanced ROS in comparison to wildtype worms, implying that ROS reduction in the *xdh-1* mutant background is α-syn specific. Since ROSs contribute to oxidative stress, mitochondrial dysfunction, and ultimately neuronal death, the decrease in ROS observed in *xdh-1(ok3134)* mutants could contribute to neuroprotection. *xdh-1* mutant worms lacking α-syn exhibited enhanced ROS production at 2.5 h, indicating that changes in ROS production due to XDH/XO activity are α-syn specific.

### 3.5. A Target of ADR-2 Editing, wht-2, Interacts in a Network with xdh-1 to Mediate PD-Associated Pathologies

Although XDH-1 is downregulated in the absence of ADR-2 function, it is not edited [43]; thus, it is likely that the decrease in *xdh-1* expression in *adr-2* mutants is due to the interaction of XDH-1 with another protein that is a substrate of ADR-2. To determine which gene is edited by ADR-2 that interacts with XDH-1, a genetic interaction network that includes 18,183 predicted interactions in *C. elegans* [49] was cross-referenced with genes edited by ADR-2 [43]. Specifically, genes that interact with XDH-1 and are also targets of ADR-2 were identified. Only one gene, *wht-2*, encoding the worm ortholog of the human ABCG2 transporter, met these criteria. Consequently, *wht-2* has previously been associated with gout [34], a condition that results from high levels of serum urate [50], an end-product of hypoxanthine or xanthine catabolism by XDH-1. Thus, *wht-2* was deemed a promising link between ADR-2 and XDH-1.

**Figure 4:**
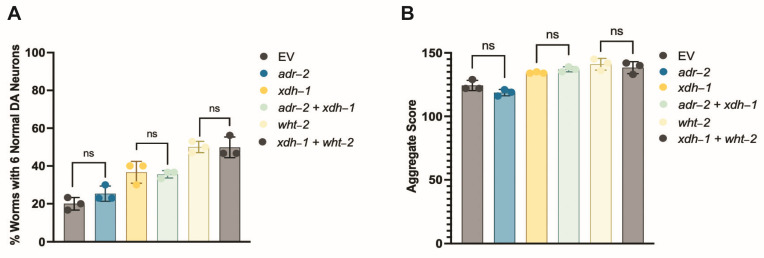
*xdh-1*, *wht-2*, and *adr-2* comprise a genetic regulatory network that affects PD pathologies in a tissue-specific manner. (**A**) Synchronized worms were scored for dopamine neurodegeneration seven days post hatch. Knockdown of *xdh-1* and *xdh-1 + ad--2* indicate that ADR-2 does not impact XDH-1 activity, while knockdown of *wht-2* + *xdh-1* indicates that XDH-1 does not regulate WHT-2 activity. These experiments were completed via RNAi knockdown in dopaminergic neurons of *C. elegans*, expressing α-syn and GFP, separately, under control of the P*_dat-1_* promoter (three independent experiments, *n* = 30 per independent groups; *N* = 3; *n* = 30). EV = empty vector control. Significance was obtained using a one-way ANOVA with a Tukey’s post hoc test; ns > 0.05. (**B**) The same experimental paradigm was performed in worms expressing an α-syn::GFP fusion protein in the body wall muscles. Synchronized two-day old worms were scored for aggregate size and number, which were combined to generate an overall aggregate score. The relation between *adr-2*, *xdh-1*, and *wht-2* in the body wall match that shown in dopaminergic neurons, implying that *wht-2* is the rate-limiting member of this system (three independent experiments, *n* = 30 per independent groups; *N* = 3; *n* = 30). EV = empty vector control. A One-way ANOVA with a Tukey’s post hoc analysis was employed, ns > 0.05.

To further elucidate the potential relationship between ADR-2, XDH-1, and WHT-2, RNAi of all three genes was performed in dopaminergic neurons and evaluated for neurodegeneration seven days post hatch. *xdh-1* knockdown was completed as proof of principle—as expected, roughly double the number of worms with decreased *xdh-1* expression in dopaminergic neurons had six intact neurons seven days post hatch, compared to those in the control group (Figure 4A). However, knockdown of *adr-2* in worms either expressing α-syn and GFP in dopaminergic neurons or in the body wall muscles did not lead to a significant phenotype; this is surprising, since 169 genes are differentially expressed upon the loss of *adr-2*, including *xdh-1*. Further, it has been shown that loss of ADAR function in neurons is detrimental in other species; for example, *ADAR2* null mice exhibit seizures and die prematurely [14]. To further explore this result, worms with GFP (Figure S4A) and those with α-syn and GFP (Figure S4B) expressed under the *dat-1* promoter were crossed to *adr-2* knockout worms. Upon analysis, it was confirmed that loss of ADR-2 is neither protective, nor detrimental, to dopamine neuron health in both the presence and absence of α-syn in *C. elegans.* Further, *xdh-1* and *adr-2* were simultaneously knocked down in dopamine neurons, and worms showed the same level of neuroprotection as *xdh-1* knockdown alone (Figure 4A). Additionally, simultaneous “double-knockdown” of *xdh-1* and *wht-2* in dopamine neurons mirrored that of *wht-2* alone. These data suggest that *adr-2*, *wht-2*, and *xdh-1* act in the same pathway to confer neuroprotection.

This experimental paradigm was repeated in the protein misfolding assay with worms expressing α-syn and GFP in the body wall muscles. Knockdown of *adr-2* did not alter protein misfolding (Figure 4B), while RNAi of *xdh-1* and *adr-2* + *xdh-1* lead to comparable changes in protein misfolding. Similarly, knockdown of *wht-2* and *xdh-1* + *wht-2* had the same results. These data indicate that these genes comprise a regulatory network that impacts PD pathologies in a tissue-specific manner. Upon editing by ADR-2, the structure of WHT-2 is best suited for the export of uric acid, a product of purine catabolism by XDH. In contrast, in the absence of editing, the structure of WHT-2 limits uric acid export. As a result, there is a downregulation of XDH transcription to limit the amount of uric acid produced and maintain cellular homeostasis. The elevation in uric acid is likely protective against dopaminergic neuron cell death by increasing the maturation of oligomeric to aggregated α-syn, the less cytotoxic form of this protein.

### 3.6. A-to-I RNA Editing of wht-2 Is Predicted to Alter WHT-2 Protein Structure

**Figure 5:**
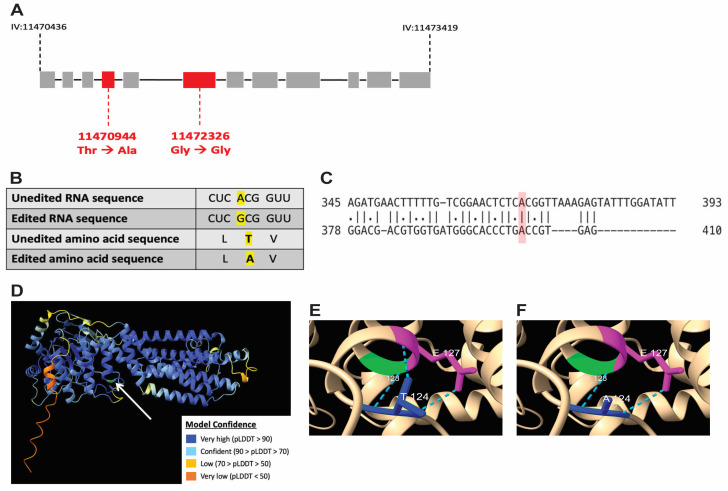
RNA editing of *wht-2* leads to a non-synonymous amino acid substitution that is predicted to alter protein folding. (**A**) Editing sites in the *wht-2* gene. *wht-2* consists of 12 exons located at genomic position IV: 11470436 to IV: 11473419 in the *C. elegans* genome. Two bases in the coding mRNA are edited, with one resulting in an amino acid substitution upon translation. (**B**) Comparison of the site (highlighted in yellow) of the *wht-2* RNA and amino acid sequences, respectively, in the presence and absence of editing at IV: 11470944. (**C**) Pairwise alignment between *C. elegans wht-2* (top) and human *ABCG2* (bottom) DNA sequences. The identified editing site, IV: 11470944 in the *C. elegans* genome, highlighted in red, is located in a region conserved between the *C. elegans* and human genomes. (**D**) The complete structure of the unedited WHT-2 protein. The arrow indicates residue 124 where threonine is switched to alanine when RNA is edited, outlined in green. The key indicates AlphaFold model prediction confidence. (**E**,**F**) Changes in protein structure at residue 124 when WHT-2 is not edited (**D**) vs. edited (**E**). Hydrogen bonding (shown in green) and protein folding are altered because of editing. The three amino acids in the region are represented by magenta (E, glutamic acid), green (Y, tyrosine), and dark blue (T, threonine in (**E**) or A, alanine in (**F**)).

**Figure 6:**
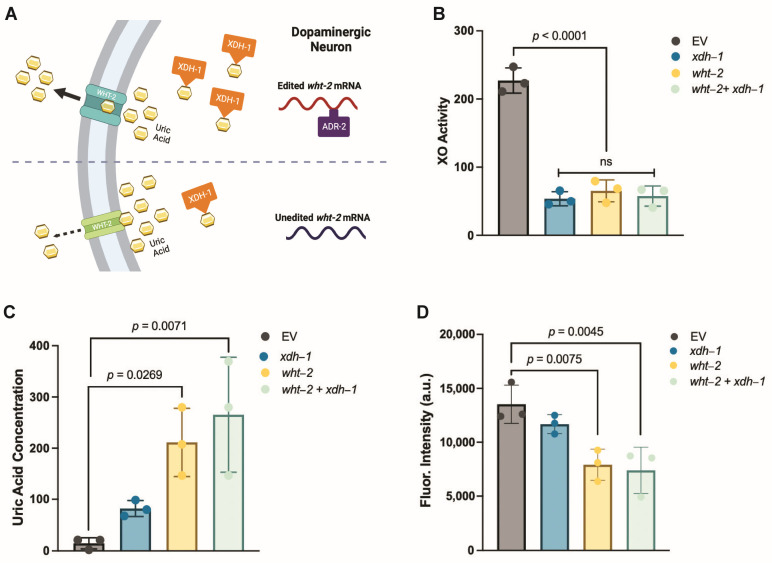
A proposed model of ADR-2, WHT-2, and XDH-1 activity. (**A**) In the presence of ADR-2, WHT-2 is edited, which allows for optimal export of uric acid, a product of purine catabolism by XDH-1. In the absence of editing by ADR-2, the structure of WHT-2 is altered, such that uric acid cannot efficiently be exported out of the cell. To maintain cellular homeostasis, there is a downregulation of *xdh-1* transcription. Created with Biorender.com. (**B**) RNAi of *xdh-1*, *wht-2*, and *wht-2 + xdh-1* was performed in *C. elegans* (UA49), expressing an α-syn::GFP fusion protein in the body wall muscles, and an enzymatic, calorimetric assay was used to measure xanthine oxidase (XO) activity two days post hatch. Three biological replicates and two technical replicates were completed for all worm strains. After 1 h, differences in activity between experimental groups were negligible, but all were significantly lower than empty vector (EV) control worms, indicating that *wht-2* acts upstream of *xdh-1*. Significance was obtained using a one-way ANOVA with a Tukey’s post hoc test and are represented by exact *p* values (*N* = 3; *n* = 30). (**C**) The same RNAi experiments were performed, and uric acid levels were measured using an enzymatic, fluorescent assay. Three biological replicates and two technical replicates were completed for all worm strains, and uric acid levels were recorded 10 min after enzymatic treatment. Two-day old worms in which *wht-2* and *wht-2* + *xdh-1* had similar levels of uric acid were significantly higher than in those in the EV control group. Worms in which *xdh-1* was knocked down had higher levels of uric acid than EV control worms and lower levels than in worms in which *wht-2* was knocked down, although levels were not significant. Significance was determined by a one-way ANOVA with a Tukey’s post hoc test and are represented by exact *p* values (*N* = 3; *n* = 30). Thus, uric acid levels are putatively controlled by WHT-2 function. (**D**) The same RNAi experimental paradigm was completed, and worms were treated with a specialized fluorescein to measure reactive oxygen species (ROS). Three biological replicates and two technical replicates were completed for all worm strains. After 2.5 h, animals with reduced *wht-2* and *wht-2 + xdh-1* expression had comparable levels of ROS, which were lower than those in the EV control group. Animals with reduced *xdh-1* expression had lower ROS levels than those in the EV control group, but higher ROS levels of those with reduced *wht-2* expression, although levels were not significant. Significance was indicated using a one-way ANOVA, with a Tukey’s post hoc test, and they are represented by exact *p* values (*N* = 3; *n* = 30). These data negatively correlate with uric acid levels, indicating that higher levels of uric acid are associated with lower ROS production.

To further analyze the impact of RNA editing on the interaction between WHT-2 and XDH-1, computational modeling of the presumptive unedited and edited *wht-2* gene product was performed. Previously, two sites in the *wht-2* gene were identified as targets of RNA edited by ADARs, located at IV: 11470944 and IV: 11472326 in the *C. elegans* genome [39]. Because inosine is treated as guanosine by translational machinery, the RNA sequence of the *wht-2* gene at the identified sites was changed to guanosine to reflect editing. The resulting sequence was translated, and the non-edited and edited amino acid sequences were compared. While the impact of editing at the second site is synonymous, editing at the first site results in a non-synonymous change from threonine to alanine at amino acid residue 124, located in the ABC-transporter domain of the protein (Figure 5A,B). This analysis agrees with the proposed model in which it is hypothesized that WHT-2 protein structure changes upon editing, since threonine is polar and alanine is non-polar. To determine whether the region of WHT-2 that is edited in *C. elegans* is conserved in humans, the DNA sequences of the two genes were aligned using EMBOSS Needle [40,51]. This analysis revealed that the genes are 41.7% homologous, and the region containing the editing site is highly conserved (Figure 5C). AlphaFold [41] and ChimeraX [42] were used to visualize this amino acid change and determine the potential functional impact of RNA editing at this site. The switch from threonine to alanine at WHT-2 residue 124 modifies hydrogen bonds in the region of the protein, thus affecting the overall structure of the protein (Figure 5D,E). Intriguingly, the substitution of threonine with alanine has been implicated in protein misfolding in the aggregation of proteins in PD and AD [52], in agreement with the results of *wht-2* RNAi *wht-2* in the worm body wall. These data further indicate that RNA editing impacts export of uric acid by WHT-2, and this mechanism is likely present in human PD patients. We propose a model in which editing of *wht-*2 RNA leads to optimal export of uric acid, the end-product of XDH-1 enzymatic activity. In contrast, in the absence of editing, uric acid cannot be efficiently exported, thereby resulting in XDH-1 downregulation to maintain cellular homeostasis (Figure 6A).

While it has been experimentally shown that WHT-2 is a known target of editing by ADR-2 and *xdh-1*, it is downregulated in *adr-2* mutant worms [43]. The relationship between WHT-2 and XDH-1 has not been previously described. Thus, to validate the relation between WHT-2 and XDH-1 in our model, a series of biochemical experiments preceded by RNAi of *xdh-1*, *wht-2* and *xdh-1 + wht-2* were conducted in worms expressing α-syn and GFP in the body wall muscles, two-days post hatch. XO activity was measured using an enzymatic, calorimetric assay that measures hydrogen peroxide in the sample, the bound form of superoxide anions that are the end-product of XO activity. *C. elegans*, in which RNAi was performed in *xdh-1*, *wht-2*, and *xdh-1 + wht-2*, displayed significantly reduced XO activity in comparison to control animals (Figure 6B). Further, the difference in XO activity between all three experimental groups was negligible, very strongly indicating that WHT-2 acts upstream of XDH-1.

The same RNAi experimental paradigm was performed, and levels of uric acid were measured using an enzymatic, fluorometric assay. Worms with reduced *wht-2* and *wht-2 + xdh-1* exhibited comparable levels of uric acid that were higher than those with decreased *xdh-1* expression (Figure 6C). These data suggest that uric acid levels are dependent upon WHT-2 transporter activity, as uric acid was higher in animals with reduced *wht-2* expression, but there was no difference between these animals and those in which RNAi of *wht-2* and *xdh-1* was performed simultaneously. It was hypothesized that animals with reduced *xdh-1* expression would exhibit lower levels of uric acid than the control group because uric acid is the end-product of XDH-1 activity. Surprisingly, this was not observed, and worms with reduced *xdh-1* expression exhibited higher levels of uric acid than those in the control group, although the difference was not statistically significant (Figure 6C). The increased uric acid present in *xdh-1* knockdown worms could be due to a change in RNA editing or gene expression of *wht-2* when the cell detects detrimentally low uric acid levels. Nonetheless, these data provide support for the proposed relationship between XDH-1 and WHT-2.

The same series of RNAi experiments was performed one final time, and the amount of ROS present in all groups was measured. Results from these experiments show *C. elegans* with reduced *wht-2* and *wht-2 + xdh-1* exhibited comparable levels of ROS that were lower than those with decreased *xdh-1* expression (Figure 6D). Knockdown of *xdh-1* alone led to reduced levels of ROS, although this reduction was not statistically significant. This insignificance is likely due to the reduced expression of *xdh-1* in worms used for this experiment, coupled with their young age, since worms were only fed RNAi bacteria for two days. It is plausible that such levels would be significant in this strain of worms with mutant *xdh-1*, or in this strain of worms exposed to RNAi bacteria for a longer period. Importantly, there is a strong negative correlation between ROS and uric acid levels, indicating that higher uric acid levels (Figure 6C) are associated with lower ROS production (Figure 6D).

## 4. Discussion

Despite the lack of complete understanding of the causative factors of PD, it is accepted that sporadic PD cases arise because of genetic and environmental factors. Indeed, rigorous work in both the clinical and laboratory settings have convincingly connected PD with processes, such as oxidative stress and mitochondrial dysfunction. Moreover, numerous studies in various cellular and organismal models have implicated ADARs in neurological diseases, but few have directly correlated RNA editing with disease. Our present study builds upon this body of evidence by connecting ADARs to disease pathologies in multiple *C. elegans* models of PD, as well as by predicting the impact of editing at the protein level on α-syn protein misfolding and α-syn-induced neurodegeneration.

We have demonstrated that several genes regulated by ADARs are involved in α-syn misfolding and dopaminergic neuronal cell death, two pathological hallmarks of PD. We have shown that knockdown of these genes altered the misfolding of α-syn, and such modifiers of protein misfolding were most highly associated with FAD and iron ion binding. Notably, FAD and iron are essential for the function of complex II of the electron transport chain. In this step, the citric acid cycle intermediate, succinate, is oxidized to form fumarate; FAD accepts two electrons from this reaction, which are then passed to iron–sulfur clusters and subsequently to coenzyme Q. It has been shown that reactive oxygen species (ROS) can be produced at this site if electrons are delayed in the iron–sulfur clusters, or if electrons move in the reverse direction, toward complex I [53,54]. ROS have been tied to oxidative stress, as they prompt dysfunction of the ubiquitin–proteasome system, as well as mito- and autophagic-mechanisms, known contributors to PD [55]. Further, PD has been shown to be associated with increased expression of transferrin receptor 1 [56] and reduced expression of ferroportin-1 [57], leading to enhanced iron deposition. Moreover, α-syn has been shown to interact with genes associated with the import and export of iron, which detrimentally alters iron homeostasis in dopaminergic neurons [58].

When knocked down in the dopamine neurons of worms co-expressing α-syn and GFP under the control of the P_dat-1_ promoter, four presumptive modifiers of protein misfolding altered neurodegeneration: *xdh-1*, *acdh-1*, *pho-1*, *F52E1.2*, and *papl-1*. All these genes were protective when knocked down except *papl-1*, which enhanced dopaminergic neuronal cell death. Consistent with recent reports suggesting that monomeric and oligomeric α-syn is more cytotoxic than the aggregated form [59,60], the genes that were neuroprotective enhanced protein misfolding when knocked down. Further, we have shown that knockout of *xdh-1*, which conferred the strongest observed effect upon knockdown, was indeed protective in our neuronal PD model. Finally, we illustrate that WHT-2, which interacts with XDH-1 and is edited by ADR-2, appears to act in the same pathway as XDH-1 to protect dopaminergic neurons from α-syn-induced neurotoxicity. Concurrent knockdown of *xdh-1* and *wht-2* by RNAi indicates that the neuroprotection associated with loss or absence of *xdh-1* is not contingent on *adr-2* expression. Thus, we propose a model in which the absence of editing leads to ineffective WHT-2 export of uric acid, which is produced by XDH-1, in turn reducing the toxicity of α-syn and protecting dopaminergic neurons from degeneration (Figure 6).

XDH, the human ortholog of XDH-1, is responsible for the breakdown of hypoxanthine to xanthine and xanthine to uric acid, the final two steps of purine catabolism. Uric acid has been associated with neuroprotection, as it serves as a scavenger of free radicals, in turn protecting against ROS. Notably, higher serum urate has been associated with lower risk of PD and slower progression of PD [61]. Additionally, it has been demonstrated that uric acid inhibits ROS accumulation and improves mitochondrial function in rat hippocampal neurons [62]. Further, uric acid is a powerful iron chelator, and dysregulation with iron metabolism has been correlated with oxidative stress in PD [63]. Drugs that promote uric acid and iron accumulation have shown moderate success in clinical trials, but both strategies for treating PD have resulted in iatrogenic effects. For instance, uric acid accretion has resulted in kidney stones, containing uric acid crystals [25,64], and iron chelation has propagated anemia [65]. Given that both iron and uric acid are associated with XDH activity, it is plausible that this enzyme is a promising therapeutic target for PD if its activity is regulated specifically in the SNpc.

ABCG2, the human ortholog of *C. elegans* WHT-2, exports uric acid, among many other endogenous and xenobiotic substrates. Our study illustrates that RNAi of *wht-2* alone and *wht-2* and *xdh-1* simultaneously in dopamine neurons of worm expressing α-syn and GFP leads to comparable levels of neuroprotection. Thus, it is possible that XDH-1 activity is reduced to limit uric acid production when WHT-2 is dysfunctional, to avoid the deleterious effects of hyperuricemia. Both ABCG2 and XDH inhibition have been associated with hyperuricemia and gout, providing further evidence of our hypothesis that these proteins act in the same pathway. Moreover, simultaneous RNAi of both genes did not have an additive phenotypic effect in either of our PD models—additional affirmation of this conjecture.

Reports have indicated that the 3′ UTRs of many genes are edited by ADARs, as these regions contain Alu elements [66]. ABCG2 is known to be regulated by various miRNAs, including at the 3′ UTR. One study in the S1 colon cancer cell line revealed that a miRNA binds to the 3′ UTR of ABCG2, thereby decreasing expression [67]. miRNAs are known to repress translation, through various modes of regulation, including preventing translational initiation, which does not necessarily impact transcription of the target gene [68]. It is possible that editing by ADARs inhibits the binding of this miRNA to the 3′ UTR of ABCG2, leading to increased ABCG2 translation. Such events could lead to an increase in XDH activity, as more uric acid produced by XDH could be excreted out of the cell by ABCG2. However, in the absence of ADAR activity, the putative miRNA can bind to the gene encoding ABCG2, leading to reduced translation of ABCG2 and the dampening of uric acid transport out of the cell. This would potentially lead to a decrease in XDH expression to maintain cellular uric acid homeostasis (Figure 6). This prospective mechanism would explain why *xdh-1* is downregulated upon loss of *adr-2* in *C. elegans*, while *wht-2* mRNA levels are not altered [25]. Of course, this hypothesis must be validated experimentally.

It is interesting that knockdown of *xdh-1* and *wht-*2 lead to enhanced neuroprotection and increased protein misfolding. It is possible that the intracellular elevation in uric acid is protective against dopaminergic neuronal cell death by increasing the aggregation of α-syn, the less cytotoxic form of this protein. However, whether increased misfolding is associated with enhanced or diminished disease progression remains controversial. It has been shown that soluble α-syn monomers and oligomers, which precede fibril formation, are more cytotoxic than larger inclusions due to their propensity to disrupt cell membrane permeability and promote neuroinflammation [59]. Further, analysis of post mortem brain tissue has indicated that more monomers and oligomers are found in PD patients in comparison to age-matched controls [69]. Contrastingly, other researchers have associated increased protein misfolding with more severe disease [70,71]. While our results suggest that enhanced misfolding of α-syn is commensurate to increased dopaminergic neuronal health, these data are due to gene expression in the body wall muscle. RNAi of *xdh-1* was cell autonomous, and it is possible that RNAi exclusively in the body wall may yield different results. The generation of a model that expresses α-syn and GFP in the body wall muscles and is RNAi sensitive solely in the body wall muscles, in addition to more studies on the role of α-syn misfolding in PD, have the potential to resolve this issue.

This study focused on two main pathologies associated with PD: the misfolding of α-syn and progressive dopaminergic neuronal degeneration. However, this investigation does not address motor and locomotive deficits observed in PD patients [7]. Worms have a thrashing behavior in which they bend very quickly in liquid, but animals with motor loss exhibit fewer bends [72]. Additionally, *C. elegans* typically slow when they encounter a lawn of bacteria—this is known as the basal slowing response. Worms with dopaminergic neuronal loss are unable to slow in the presence of food, so they glide through the lawn [73,74], A limitation of this study is that specific motor changes associated with *adr-2*, *xdh-1*, and *wht-2* expression are not characterized. Future studies should be conducted to connect motor and locomotive phenotypes with the results reported here. Nevertheless, it should be noted that, in contrast to other widely used worm PD models that focus on quantifying behavioral changes and/or proteostasis exclusively, the specific *C. elegans* α-syn model used herein has proven highly predictive of translational outcomes pertaining to human genetic findings and evaluation of chemical modifiers when neurodegeneration is the readout [75].

Notably, loss of ADR-2 activity did not impact α-syn-induced neurodegeneration. This was surprising due to evidence that aberrant ADAR activity has detrimental neurological effects across species, including *C. elegans*. For example, it has been shown that *C. elegans* that lack editing by ADARs exhibit reduced chemotaxis [43]. However, chemotaxis in *C. elegans* is regulated by ASE sensory neurons, not dopaminergic neurons [76]. Since dopamine neurons were the only neuronal cell type evaluated in our study, it is possible that aberrant ADR-2 activity does indeed impact the health of other neuronal cell types. Further, the loss of ADR-2 did not impact aggregation of α-syn. This may be due to a lack of expression of *adr-2* in the body wall—high levels of ADR-2 activity have been reported in the *C. elegans* nervous system specifically [22,25], but expression patterns in other tissues have not been extensively studied. It is also possible that the absence of observable phenotypes attributable to *adr-2* deletion in neurons and body wall muscles expressing α-syn may be an additive effect of changes in expression of all targets of ADARs—simply put, some genes may lead to enhanced neurodegeneration or misfolding, or vice versa, cumulatively leading to no net change.

Here, we describe a functional relationship between ADR-2, WHT-2, and XDH-1 that impacts PD-associated pathologies. Further, we describe how RNA editing by ADARs affects the folding of the WHT-2 protein, presumably changing *xdh-1* expression. While additional work is needed to further elucidate this mechanism, the targeting of ABCG2, the human ortholog of WHT-2, by ADARs, may serve as a promising therapeutic strategy for the treatment of PD. Because ADARs modify many genes in different tissues, the targeted altering of editing or edited substrate proteins, opposed to a complete abolishment of editing, is ostensibly a better approach toward therapeutic intervention. Nonetheless, RNA editing represents an exciting area of inquiry, with substantial implications for biomedical research. The foundational studies on ADAR function in *C. elegans* have established this system as an exceptional model for investigation of these transformative enzymes as putative modifying factors in neurological diseases such as PD [20,23,77]. Moreover, the cost-effective and expedient nature of *C. elegans* as a system to accelerate discovery remains largely underutilized towards translational goals, but it is expanding as an outcome of the increasingly proven preclinical utility of worm disease models [75,78,79].

## Figures and Tables

**Table 1:** Summary of genetic crosses completed to generate new worm strains used in this study.

Strain	Genotype	Strains Crossed
UA455	*xdh-1(ok3134)*; *baIn11*[P*_dat-1_*::α-syn, P*_dat-1_*::GFP]	RB2379 × UA44
UA456	*xdh-1(ok3134)*; *vtIs7* [P*_dat-1_*::GFP]	RB2379 × BY250
UA457	*adr-2(ok735)*; *vtIs7*[P*_dat-1_*::α-syn, P*_dat-1_*::GFP]	RB886 × BY250

**Table 2:** *C. elegans* genes screened via RNAi for changes in α-synuclein protein misfolding. The 14 genes that led to the greatest change in protein misfolding and were considered for further analysis are shown in red. All other genes that were analyzed for changes in α-synuclein misfolding and did not elicit a phenotype are displayed in black. Genes are written in italics in accordance with proper *C. elegans* nomenclature.

Genes			
*acdh-1*	*ctc-3*	*hpo-29*	*papl-1*
*acox-1*	*ctl-2*	*hsp-12.3*	*pept-1*
*acox-1.4*	*cyp-13A12*	*hsp-12.6*	*pho-1*
*act-5*	*cyp-25A2*	*ifp-1*	*srsx-33*
*ads-1*	*dhs-28*	*K02F6.8*	*T05C3.2*
*asns-2*	*dod-17*	*K03H1.5*	*T22F3.7*
*C06G8.3*	*dsc-4*	*K08D8.6*	*ttll-9*
*C29F3.7*	*ets-4*	*K10C2.6*	*ugt-16*
*C29F3.7*	*ets-9*	*K10D11.3*	*ugt-37*
*C31H5.6*	*F09F7.5*	*lec-10*	*ugt-44*
*ccpp-6*	*F11C7.2*	*lipl-1*	*xdh-1*
*chil-13*	*F15E6.6*	*lipl-7*	*Y32F68.1*
*clec-3*	*F21D5.3*	*ltah-1.2*	*Y44A6D.5*
*clec-4*	*F41E7.6*	*metr-1*	*Y47H10A.5*
*clec-41*	*F52E1.2*	*mth-1*	*Y48A6B.7*
*clec-42*	*fmo-5*	*nduo-1*	
*crn-6*	*folt-2*	*nep-17*	
*ctc-2*	*H43E16.1*	*nep-22*	

**Table 3:** Summary of hits of RNAi screen. Genes that produced a phenotype upon RNAi in dopaminergic neurons include xdh-1, acdh-1, pho-1, F52E1.2, and papl-1. The gene name, human orthologs [45], differential expression in adr-2 mutant worms [25], change in protein misfolding upon RNAi (Figure 1A), change in neurodegeneration upon RNAi (Figure 2C), and a general description of each gene are provided [46,47]. Significance of change in neurodegeneration is represented by exact *p* values.

Gene Name	Human Ortholog(s)	Differential Expression in *adr-2* Mutant	Change in Protein Misfolding	Change in Dopamine Neuron Degeneration	Description
*xdh* * - * *1*	*XDH*	Downregulated	+8.55%	Protective (*p* = 0.0002)	Catalyzes the final two steps of purine catabolism; low oxidase activity toward aldehydes; produces ROS.
*acdh* * - * *1*	*ACADSB*	Downregulated	+14.47%	Protective (*p* = 0.0164)	Promotes acyl-CoA dehydrogenase activity; involved in innate immune response.
*pho* * - * *1*	*ACP2*, *ACPT*	Downregulated	+10.92%	Protective (*p* = 0.0198)	Converts orthophosphoric monoesters to alcohol and phosphate via hydrolysis; encodes phosphatase activity.
*F52E1.2*	*CLEC4A*, *CLEC4C*, *CLEC4D*, *CLEC4E*, *CLEC6A*, *ASGR1*	Upregulated	+11.93%	Protective (*p* = 0.0452)	Promotes carbohydrate binding activity; involved in cell signaling, adhesion, glycoprotein degradation and production, inflammation, immune response.
*papl* * - * *1*	*ACP7*	Downregulated	+9.195%	Enhanced (*p* = 0.0361)	Promotes acid phosphatase activity; enables metal ion binding.

## Data Availability

All study data are included either in the main text of the article or in the Supplementary Materials.

## Supplementary Materials

The following supporting information can be downloaded at: https://www.mdpi.com/article/10.3390/jdb11020020/s1, Figure S1: Analysis of neuronal health in worm populations upon RNAi of candidate genes in all tissue types excluding neurons; Figure S2: qPCR of α-syn in worms independently expressing α-syn and GFP under control of the P*_dat-1_* promoter, with and without functional *xdh-1* seven days post-hatch; Figure S3: Knockout of *xdh-1* in P*_dat-1_*::GFP worms does not impact dopaminergic neuron health; Figure S4: Knockout of *adr-2* does not impact dopaminergic neuronal health.
