# Supplementary material for: Attenuation of Dopaminergic Neurodegeneration in a C. elegans Parkinson’s Model through Regulation of Xanthine Dehydrogenase (XDH-1) Expression by the RNA Editase, ADR-2
Source: J Dev Biol. 2023 May 22;11(2):20. doi: 10.3390/jdb11020020 (PMC10204437; doi:10.3390/jdb11020020)
Supplement: Supplementary file 1 [file jdb-11-00020-s001.zip › jdb-2307954-supplementary.pdf]

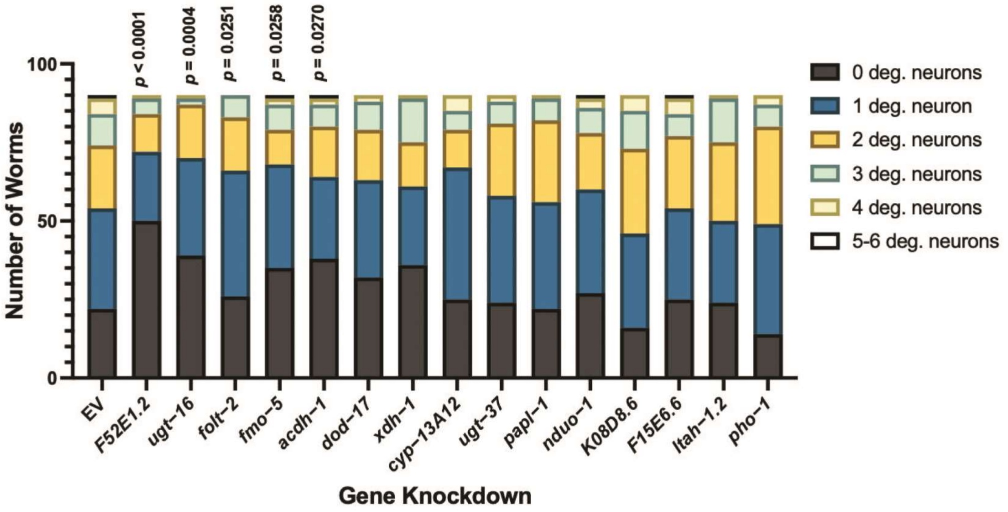

Figure S1. *Analysis of neuronal health in worm populations upon RNAi of candidate genes in all tissue types excluding neurons.* Candidate genes were knocked down in all cell types, excluding neurons of *C. elegans*, expressing α-syn and GFP under control of the P_{dat-1} promoter. Subsequent scoring of dopamine neuron health and worm population analysis indicate that RNAi of *F52E1.2* was robustly protective. Knockdown of *ugt–16* was substantially protective, while RNAi of *folt–2*, *fmo–5*, and *acdh–1* were significantly protective against α-syn-induced dopamine neurodegeneration. No genes enhanced dopamine neuron death when knocked down. Synchronized worms were analyzed as seven-day old adults (three independent experiments, $n = 30$ per independent groups). Chi-square analysis was performed, where each candidate gene knockdown value was compared to EV and are represented by exact p values (three independent experiments, $n = 30$ per independent groups; $N = 3$; $n = 30$).

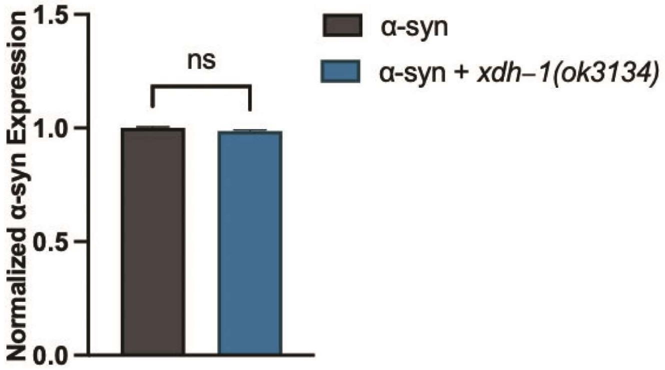

Figure S2. qPCR of α-syn in worms independently expressing α-syn and GFP under control of the P_{dat-1} promoter, with and without functional xdh–1 seven days post hatch. α-syn expression was normalized to three housekeeping genes: *snb–1*, *tba–1*, and *ama–1*. Three independent experiments, $n = 30$ per independent groups, three technical replicates. Worms with the *xdh–1(ok3134)* mutation did not show a difference in α-syn expression compared to those with wildtype *xdh–1* in the α-syn background. mRNA data analyzed by Student's t-test and presented as mean +/− SEM of three biological replicates, with three technical replicates each; at least 100 animals were used for each replicate. These data were normalized to the α-syn control.

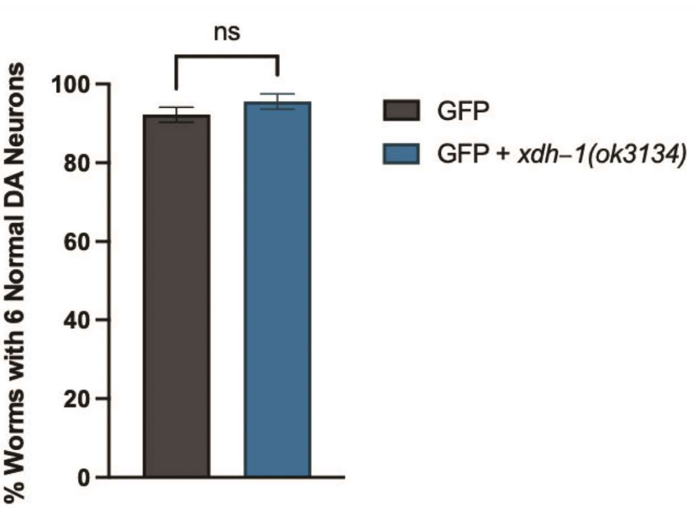

Figure S3. *Knockout of xdh-1 in P_{dat-1}::GFP worms does not impact dopaminergic neuron health*. Animals with wildtype or mutant *xdh–1* were crossed to isogenic worms expressing only GFP in dopamine neurons under control of P_{dat-1}. The four CEP and two ADE dopaminergic neurons were analyzed for the presence or absence of intact cell bodies and axonal processes in synchronized worms seven days post hatch (three independent experiments, n = 30 per independent groups). Worms with the *xdh–1(ok3134)* mutation do not show a change in dopaminergic neuronal health. Student's t-test, where GFP + *xdh–1(ok3134)* was compared to GFP alone (three independent experiments, n = 30 per independent groups; N = 3; n = 30); ns > 0.05

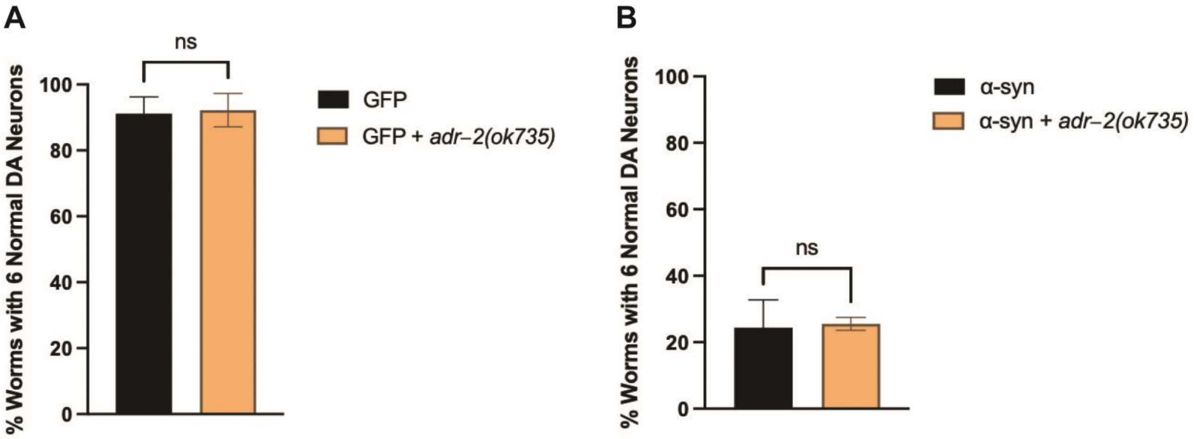

Figure S4. *Knockout of adr–2 does not impact dopaminergic neuronal health*. (**A**) Animals with wildtype or mutant *adr–2* were crossed to isogenic worms expressing either GFP in dopamine neurons, or GFP + α-syn, independently, under control of the P_{dat-1} promoter. The four CEP and two ADE dopaminergic neurons were analyzed for the presence or absence of intact cell bodies and axonal processes in synchronized worms seven days post hatch (three independent experiments, n = 30 per independent groups). Worms with the *adr–2(ok735)* mutation do not show a change in dopaminergic neuronal health in both the absence (**A**) or presence (**B**) of α-syn. Student's t-test, where GFP + *adr–2(ok735)* was compared to GFP alone (three independent experiments, n = 30 per independent groups; N = 3; n = 30) in the absence (**A**) or presence (**B**) of α-syn; ns > 0.05.
